# Clinical results of a surgical technique using endobuttons for complete tendon tear of pectoralis major muscle: report of five cases

**DOI:** 10.1186/1758-2555-3-20

**Published:** 2011-09-28

**Authors:** Yoshiyasu Uchiyama, Seiji Miyazaki, Tetsuro Tamaki, Eiji Shimpuku, Akiyoshi Handa, Hiroko Omi, Joji Mochida

**Affiliations:** 1 Department of Orthopaedic Surgery, Surgical Science, Tokai University School of Medicine, Kanagawa, Japan; 2 Department of Judo and Kendo, Tokai University School of Physical Education, Kanagawa, Japan; 3 Department of Regenerative Medicine, Division of Basic Clinical Science, Tokai University School of Medicine, Kanagawa, Japan

## Abstract

**Background:**

We herein describe a surgical technique for the repair of complete tear of the pectoralis major (PM) tendon using endobuttons to strengthen initial fixation.

**Methods:**

Five male patients (3 judo players, 1 martial arts player, and 1 body builder) were treated within 2 weeks of sustaining complete tear of the PM tendon. Average age at surgery and follow-up period were 28.4 years (range, 23-33) and 28.8 months (range, 24-36). A rectangular bone trough (about 1 × 4 cm) was created on the humerus at the insertion of the distal PM tendon. The tendon stump was introduced into this trough, and fixed to the reverse side of the humeral cortex using endobuttons and non-absorbable suture. Clinical assessment of re-tear was examined by MRI. Shoulder range of motion (ROM), outcome of treatment, and isometric power were measured at final follow-up.

**Results:**

There were no clinical re-tears, and MRI findings also showed continuity of the PM tendon in all cases at final follow-up. Average ROM did not differ significantly between the affected and unaffected shoulders. The clinical outcomes at final follow-up were excellent (4/5 cases) or good (1/5). In addition, postoperative isometric power in horizontal flexion of the affected shoulder showed complete recovery when compared with the unaffected side.

**Conclusions:**

Satisfactory outcomes could be obtained when surgery using the endobutton technique was performed within 2 weeks after complete tear of the PM tendon. Therefore, our new technique appears promising as a useful method to treat complete tear of the PM tendon.

## Background

The pectoralis major (PM) muscle is a powerful adductor, flexor, and internal rotator of the shoulder, and contributes considerably to upper-body power and motor capacity. Athletes who lose all PM muscle function following a complete tear of this muscle, even unilaterally, risk losing their career. Therefore, complete tear of PM is considered a severe, incapacitating sports injury.

PM injury can be categorized roughly into three types: type 1, limited to contusions and sprain of the muscle with no tear; type 2, partial tears of muscle fibers or tendon; and type 3, severe tears. Type 3 injuries are further subdivided as follows into three grades by location of the tear: grade 1, muscle belly; grade 2, myotendinous junction, and grade 3, distal tendon [1]. Generally, type 3 injuries of grade 2 and grade 3 are considered the best candidates for surgical repair, and the remaining injury types are likely to be initially managed conservatively with the anticipation of natural healing [2,1]. In contrast, natural healing cannot be expected for the complete- or near-complete tears, so surgical repair should be advised [3-5]. Moreover, non-surgical treatment for the complete tear of the PM muscle also fails to correct the contour of the anterior axilla, causing a poor cosmetic result.

Several surgical techniques have been reported for complete tears of PM [3,6-8]. However, we believe that these techniques do not provide strong fixation between PM and the humerus. As described above, PM is the major muscle of the upper body and contributes considerably to motor capacity. Moreover, during sports, huge strains are placed on the insertion of PM. Athletes may therefore be wary regarding re-tear, and this may adversely affect their sports performance. In order to address these concerns among competitive athletes, we considered it necessary to create a surgical technique with strong initial fixation of the insertion of the PM tendon.

Here, we report a surgical technique for the repair of complete tear of the PM tendon, i.e., grade 3, type 3 injury, in which strong initial fixation is achieved using endobuttons through a rectangular bone trough. We discuss the clinical outcome of five athletes treated with this technique.

## Methods

### Subjects

The participants of this study were five young male patients who were diagnosed as having complete tear of the PM tendon and underwent the present primary repair surgery at our hospital or another affiliated hospital from 2002 to 2007. They comprised two elite judokas (judo players), one collegiate judoka, one recreational body builder, and one competitive mixed martial arts player. In three cases, the tears were on the right side and in two they were on the left; they were on the dominant side in three cases. The average age at surgery was 28.4 years (range, 21-33), and the average duration of follow-up was 30.2 months (range, 24-36) (Table 1). These characteristics are summarized in Table 1. None of the subjects reported any history of anabolic steroid use. The injury occurred while playing judo in three cases (the three judokas) and during bench press exercise in the other two cases. All injuries happened during the intensive eccentric contraction phase of PM.

**Table 1:** Detail of Patients

Number of Patients	Sex	Age (years)	Dominant/Affected shoulder	Sports	Sports level	Period of injury to surgery (days)		Physical Findings	
							
							Swelling	Ecchymosis	Deformity
1	Male	26	Right/Right	Judo	Elite	10	++	+	++
2	Male	31	Right/Right	Judo	Elite	5	++	++	-
3	Male	21	Right/Left	Judo	Collegiate	8	++	++	++
4	Male	33	Right/Left	Bodybuilder	Recreational	12	+	++	+
5	Male	31	Right/Right	Martial art	Competitive	11	+	+	-

Average		28.4				9.2			

2+; sevire, 1+; mild, -; none

### Diagnosis

**Figure 1:**
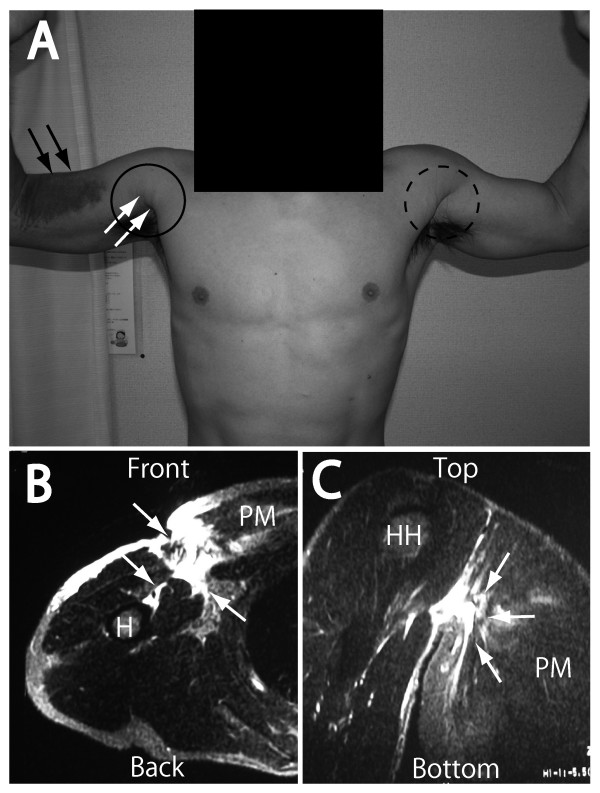
**Diagnosis of acute tendon tears of the pectoralis major muscle in patient 2**. Clinical photograph of the patient shows ecchymosis over the anterior aspect of the right arm 2 days after injury (A, black arrows) and loss of the axillary fold (A, white arrows inside the black circle; the dotted black circle indicates the normal left side). Magnetic resonance imaging (MRI) with axial (B) and coronal (C) views; spin echo T2-weighted image shows disruption of the tendon of the pectoralis major (PM) muscle. The area of high signal intensity has a fluid-fluid level (B and C, white arrows), which was thought to indicate a loculated hematoma. PM, pectoralis major; H, humerus; HH, humeral head.

In patients with acute complete tear of the PM tendon, physical examination revealed swelling and ecchymosis of the anterior axilla and upper arm area (Figure 1A; black arrows). Some objective weakness of adduction or internal rotation was common. Deformity with medial retraction of the PM muscle belly was not always evident in patients in acute phase, particularly in those with well-developed muscles. However, 1-2 weeks after injury, abduction of the arm created a "webbed" appearance at the anterior axilla as the torn distal portion of the PM muscle retracted into the chest wall; this was less apparent with the arm at the side. With shortening of the PM muscle, the deformity became obvious as the muscle retracted medially, and loss of contour of the anterior chest wall was sometimes observed (Figure 1A; white arrows). This could be detected by comparison of affected and normal sides (solid and dotted circle). In all cases, acute complete tear of the PM tendon could be confirmed by MRI (Figure 1B, C; white arrows). The physical findings of all subjects are summarized in Table 1.

### Surgical Technique

**Figure 2:**
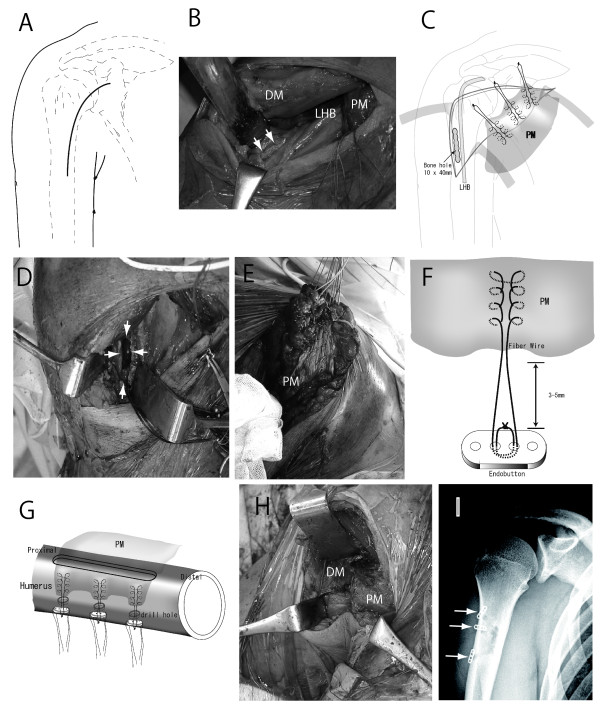
**Schematic drawings and intraoperative findings of the present surgical technique**. A, the modified anterior axillary incision used in our procedure. B, anatomic attachment of the pectoralis major (PM) muscle (white arrows). C and D, a rectangular bone trough (about 10 mm width × 40 mm height square, and about 20 mm depth through the cortical bone, white arrows in D) was made in the proximal humerus at the anatomic insertion of PM. Three sets of horizontal and vertical grasping stitch (Krackow sutures technique) were inserted using non-absorbable sutures (Fiberwire N°2; Arthrex, Inc, Naples, FL, USA) to adequately grasp the posterior surface (muscle fascia) of the PM muscle (C, E, and F). Endobuttons (Acufex; Smith & Nephew, Inc., Andover, MA, USA) were secured at the ends of the non-absorbable sutures (F). The tendon stump about 20 mm was introduced into this trough in order to transfix it to the other side of the cortex using endobuttons (I; postoperative X-ray, white arrows) and non-absorbable sutures (G; schema of transfixion technique and H; intraoperative view after transfixion technique). DM, deltoid muscle; PM, pectoralis major; LHB, long head of biceps.

Patients were positioned in the semi-beach chair position with the affected arm draped free. An axillary incision line was extended distally along the proximal humerus and proximally along the PM (Figure 2A). The deltopectoral interval was easily found and the deltoid muscle was retracted laterally (Figure 2B). Red-brown exudate fluid often filled the gap corresponding to the tear in PM. The muscle belly could be easily freed from the anterior chest wall medially and mobilized freely, and allowing it to be pulled back laterally to its anatomic attachment (Figure 2B, white arrows). A rectangular bone trough (about 10 mm width × 40 mm height square, and about 20 mm depth through the cortical bone: Figure 2C and white arrows in D) was created in the proximal humerus just lateral to the long head of the biceps tendon at the anatomic insertion of PM (Figure 2C, D. Three sets of horizontal and vertical grasping stitches (the Krackow suture technique) were performed using non-absorbable sutures (Fiberwire N°2; Arthrex, Inc, Naples, FL) to adequately grasp the posterior muscle surface (muscle fascia) of PM (schema in Figure 2C and photograph in Figure 2E). Endobuttons (Acufex; Smith & Nephew, Inc, Andover, MA, USA) were secured at the end of the non-absorbable sutures (Figure 2F), and the tendon stump was introduced into the trough to be transfixed to the other side of the cortex using endobuttons and sutures (Figure 2G, H and postoperative X-ray in Figure 2I). The stump of PM tendon was introduced about 20 mm in bone. The wound was thoroughly irrigated and closed, and the axillary deformity was found to have been corrected thereafter.

### Postoperative Care

The operated arm was held at 10 degrees flexion and 40 degrees internal rotation in a sling/immobilizer for 4 weeks after surgery. During this period, active use of the upper extremity below the elbow was encouraged. Thereafter, pendulum exercise and passive self-assisted and active isometric exercises in elevation, abduction, and external rotation were gradually introduced. During this time, active isometric exercises in internal rotation were prohibited to protect PM. Muscle strength exercises were started at 9 to 10 weeks after surgery. Moderate muscle strength exercises were allowed at 3 months after surgery, and a return to pre-injury or vigorous athletic activity was allowed 6 months after the recovery of muscle strength.

### Clinical Evaluations and Diagnostic Imaging at Final Follow-up

Clinical assessment included the following: rate of re-tear on MRI (1.5 Tesla MRI System, Gyroscan ACS/NT; Philips Medical Systems, Best, Netherlands) with a gradient system and T2-weighted fat suppression fast spin echo; range of motion of the shoulder (by manual measurement using a protractor); and isometric strength testing at final follow-up. Isometric strength testing was assessed with the use of a Commander Power Track II (JTECH Medical, Salt Lake City, UT, USA) in all patients. Tests were performed with the patients in the supine position on a bench with the shoulder in 90° of forward elevation. Straight flexion, extension, internal rotation, external rotation, horizontal adduction, and horizontal abduction were tested. The patient was asked to resist each type of motion for 5 seconds, and the mean of three tests was recorded as the strength of the shoulder. In addition, the thickness of the unaffected and affected PM tendon was measured by postoperative axial MRI and CT was performed to clearly confirm bone union in the rectangular bone trough to the humeral insertion of PM. The clinical outcome of treatment at the final follow-up was graded as excellent, good, fair, or poor according to the criteria of Bak et al. [9] (Table 2).

**Table 2:** The Criteria for Evaluation of the Result of the Treatment at Final Follow-up^2^

Results	Criteria
Excellent	The patient was pain free, had a full range of motion, no cosmetic complaints, had symmetrical manual adduction strength assessment or less than 10% isokinetic strength loss, and had returned to previous activities without restrictions.
Good	The patient had only slight functional impairment with only restrictions in movement or strength, and without cosmetic complaints with symmetrical manual adduction strength, or less than 20% isometric deficit.
Fair	There was an impairment of function which affected return to desired activity, that is, pain or weakness on activity, or if the cosmetic result was unsatisfactory.
Poor	in cases of tretment failure, that is, for non-surgical treatment if an operation was required after a minimum of 16 weeks after the injury

### Statistical Analysis

Data were analyzed using Statview software (Abacus Concept Inc., Berkeley, CA, USA). The Wilcoxon signed rank test was performed to assess the difference in range of motion (ROM), isometric strength, and thickness of the PM tendon postoperatively in the affected and unaffected shoulders.

## Results

**Figure 3:**
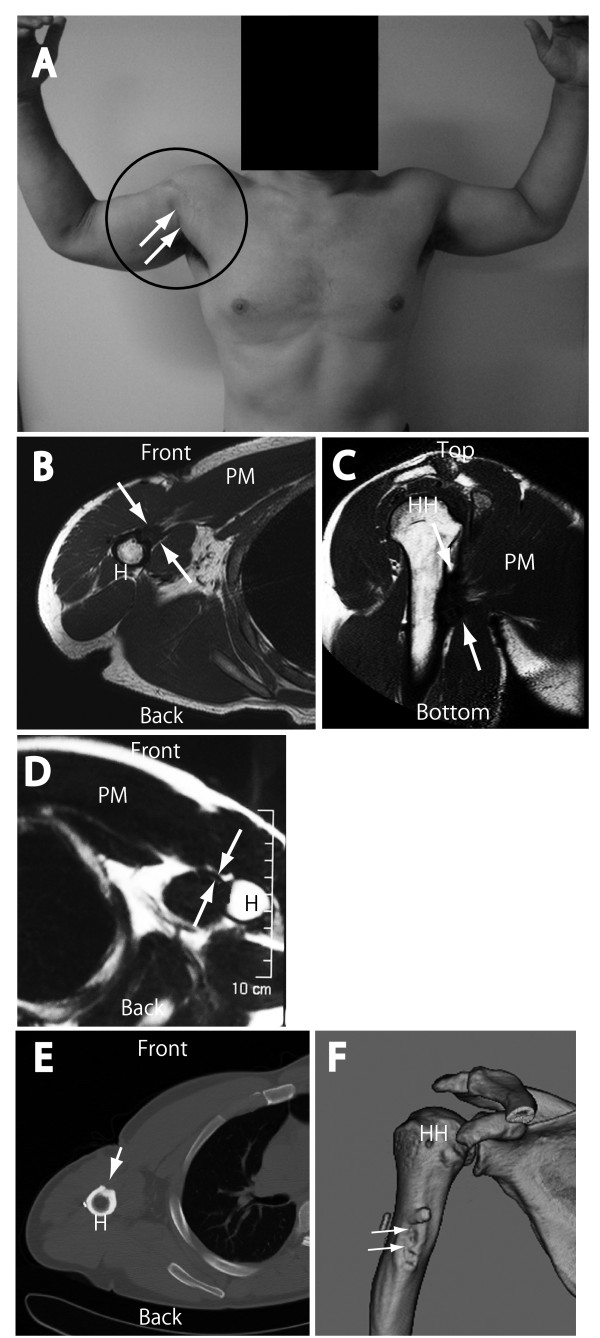
**Postoperative external appearance and imaging in patient 1**. The postoperative photograph shows that the loss of the axillary fold (contour of the anterior chest wall) had recovered 30 months after injury (A, white arrows inside the black ring). Magnetic resonance imaging (MRI) with axial (B) and coronal (C) views; spin echo T2-weighted image shows continuation of the tendon between the pectoralis major muscle and humerus (B and C, between white arrows). The thickness of the affected PM tendon (B, between white arrows) was significantly greater than that of the unaffected PM tendon (D, between white arrows). Furthermore, the rectangular bone trough was ossified as determined by computed tomography (CT) (E, white arrow) and three-dimensional CT (F, white arrows), and this bone union indicates that the reconstructed tendon was fixed strongly to the humerus. PM, pectoralis major; H, humerus; HH, humeral head.

None of the five cases showed evidence of re-tear. Thirty months after of surgery, loss of contour of the anterior chest wall was no longer visible in case 1 (Figure 3A, white arrows in the circle), and MRI findings also showed continuity of the PM tendon (Figure 3B; axial view, D; coronal view). Similar trends were consistently observed in other cases at final follow up. Moreover, axial (Figure 3E) and three-dimensional (Figure 3F) CT findings showed complete bone union to the rectangular bone trough. This bone union suggested that the reconstructed tendon was fixed strongly to the humerus. Interestingly, the thickness of the affected PM tendon (Figure 3C, between white arrow) was significantly greater than that of the contralateral unaffected PM tendon (Figure 3D, between white arrow, 1.96 ± 0.27 mm vs. 8.94 ± 1.81; p < 0.01; Table 3).

**Table 3:** The thickness of PM tendon in postoperative axial MRI

	Unaffected side	Affected side	
Case	(mm)	(mm)	p
1	1.7	11.3	
2	1.8	7.8	
3	2.2	8.6	
4	1.8	6.8	
5	2.3	10.2	

Avg.	1.96	8.94	< 0.01

There was no significant difference between the affected and unaffected shoulder with regard to mean ROM at the final follow-up (Table 4). The mean postoperative recovery of isometric power compared with the unaffected side (%) was 92.6 ± 10.7% in flexion, 96.4 ± 4.4% in extension, 99.4 ± 4.1% in external rotation with the arm along the chest, 98.8 ± 2.0% in internal rotation with the arm along the chest, 97.3 ± 5.8% in horizontal adduction, and 96.7 ± 4.8% in horizontal abduction (Table 5). Postoperative isometric power of the affected shoulder was equal to that of the unaffected shoulder. The level of clinical outcome at the final follow-up was excellent in 4/5 cases and good in 1/5 (Table 5). There were no postoperative infections, neurological complications, or implant-related complications in this series.

**Table 4:** Postoperative Active Range of Motion

Case	F/U Period (months)	Elve (°)			ER at AP (°)			IR at AP (spinus)			ER at 90°Abd (°)			IR at 90°Abd (°)		
											
		Affected	Unaffected	p	Affected	Unaffected	p	Affected	Unaffected	p	Affected	Unaffected	p	Affected	Unaffected	p
1	30	170	170		45	50		8	6		95	100		40	40	
2	33	165	165		60	60		5	6		80	85		50	45	
3	24	170	170		70	70		6	6		95	105		30	30	
4	36	155	160		45	50		7	7		85	85		40	40	
5	28	165	165		60	60		8	7		90	90		40	40	

Avg.	30.2	165	166	ns	56	58	ns	T6.8	T6.4	ns	89	93	ns	40	39	ns

Avg.: Average, F/U: Follow-up, Postop: Postoperative, Elve.: Elevation, ER: External Rotation, AP: Anatomical Position, IR: Internal Rotation,

Flex: Flexion, Ext.: Extension, Holiz., Holizontal, Abd.: Abduction, Add.: Adduction, p value: affected vs unaffected

**Table 5:** Postoperative recovery of isometric power and function

Isometric power							
(%, Affected/unaffected side)							Functional Criteria*
**Patient number**	**Flex**	**Ext**	**ER in AP**	**IR in AP**	**Horiz Add**	**Horiz Abd**	

1	98.6	96.0	98.5	101.0	107.0	99.4	Excellent
2	102.0	94.5	105.0	99.5	95.7	96.1	Excellent
3	74.7	91.1	93.6	95.6	97.1	88.7	Good
4	91.3	97.3	101.0	98.5	91.7	98.5	Excellent
5	96.4	103.2	98.7	99.4	94.8	101.0	Excellent

Average	92.6	96.4	99.4	98.8	97.3	96.7	

Flex, flexion; Ext, extension; ER, external rotation; IR, internal rotation;

AP, anatomical position arm at side; Horiz, horizontal; Abd, abduction; Add, adduction

## Discussion

Two types of surgical (osseous fixation) techniques for complete tear of the PM tendon have previously been reported. In one, a deep bone trough is made in the remaining tendon area on the humerus, and the sutured PM tendon is seated firmly back to its insertion site through drill holes in the humerus [10,6,11]. In our method, the original insertion area (remaining PM tendon on humeral side; about 10 mm width × 40 mm height) was determined according to the size of bone trough. In the other, the humeral insertion site of the PM tendon is decorticated (no bone trough was made). Metal suture anchors (three to five) [3], screws [12], barbed staples [13], or a washer system (two sets) [13] are placed in the original position of the torn PM tendon, and a grasping stitch is inserted. Although there are few cases of re-tear with either method and better clinical outcomes have been reported than with non-surgical treatment [7,8,12,14], we were concerned about whether the initial strength of the PM tendon fixation was sufficient. Generally, endobuttons and strong sutures (non-absorbable sutures) have been used as initial stronger fixing materials to bear considerable physical stress such as that encountered in the correction of tear of the anterior cruciate ligament of the knee [15], tear of the distal biceps tendon [16], separation of the distal tibiofibular joint [17], and separation of the acromioclavicular joint [18]. Good biomechanical and clinical results have been reported in all cases. Therefore, we believe that our technique using endobuttons and strong sutures achieved stronger fixation of the PM tendon, particularly in the initial fixation, and this may provide a greater sense of safety for athletes. In our hospital, unfortunately, we did not perform previous two types of surgical techniques for complete tear of the PM tendon, thus we could not directly compare with our endobotton technique and the previous similar techniques. However, psychological effects of strong suture would be beneficially working for the commitment to rehabilitation of our athlete patients.

In the present study, sufficient regeneration of the bone-tendon-muscle unit was observed in the enthesis of PM. The rectangular bone trough was also closed completely in all cases as shown by CT (Figure 3E, white arrows) and three-dimensional CT (Figure 3F, white arrows) at final follow-up. In the present method, the PM tendon stump was introduced through the bone tunnel and could therefore receive various factors from the bone marrow which may contribute to better tendon healing. Therefore, we hypothesized that these factors may benefit tendon healing, because better biological and biomechanical recovery of the extra-articular enthesis compared with the intra-articular end is achieved when the tendon passes through the bone tunnel (e.g., anterior cruciate ligament vs. rotator cuff) [19]. In addition, the reconstructed PM tendons were larger than those on the unaffected side, as confirmed by axial MRI (Figure 3B and 3C, between white arrows, p < 0.01). For this reason, it is considered that a lateral end of torn PM tendon was introduced deep bone trough, and more medial tendon or muscle, which is larger than a lateral end, was consequently situated at a level of humeral the insertion. Moreover, at the surgery, end of the PM muscle was inserted into the bone tunnel up to the muscle fiber portion. Skeletal muscle interstitium contains a large number of myofibroblasts which could contribute to the increase in connective tissue [20,21]. These fibroblasts could have induced tendinous structure formation in the inserted muscle fiber portion, thereby enlarging the tendon. Consequently, natural features of the bone-tendon-muscle unit could be seen in the PM. It seems that enlarged PM tendon did not affect PM muscle function because significant recovery of ROM and isometric power occurred (Tables 4 and 5).

PM tear is a rare injury with only about 200 cases reported in the literature [3], and it is typically observed in male patients (only one case has been reported in a woman) [22]. Recently, the incidence has gradually risen because of an increase in the popularity of strength training, although it remains a rare injury in global comparisons [9,6]. The cause of PM tear remains unclear. However, most cases appear to occur during strong contraction in the extension phase of PM, such as when lifting weights (particularly bench press exercise) [7], boxing, playing jiu-jitsu, and windsurfing [23]. In the present study, two of five cases were caused by bench press exercise, and the remaining three cases occurred while playing judo, and because tears were also induced during strong contraction in the extension phase of PM, this appears to be the common cause.

Generally, patients who develop a PM tear experience pain and weakness of the anterior shoulder and chest. However, because this injury is so rare, it is often misdiagnosed as a sprain or muscle strain at initial presentation and managed non-surgically [4]. If complete PM tears (grade-2 and -3 of type-3) are untreated for more than 4 weeks and surgery is delayed, the rate of functional recovery tends to decrease proportionally to the delay. In fact, it was reported that early treatment (within 3-6 weeks) is essential for debilitating injuries, to minimize the decline in function [3,14]. Therefore, prompt identification of complete PM tears is required in sports medicine.

After all, the weakness of the present study is that only five patients were included. Therefore, more operations should be a need to clarify the concrete beneficial effect of this technique, and we are currently planning to assemble this case.

## Conclusions

We have proposed a surgical technique for the repair of complete tear of the PM tendon using endobuttons, and demonstrated good clinical and radiological results in athletes performing competitive sports. When the present surgery is completed within 2 weeks after the complete tear of the PM tendon, significant functional recovery (more than 97%) was demonstrated. Our surgical technique therefore appears to have promise as a useful method of complete tearing of the PM tendon.

## List of abbreviations

PM: Pectoralis major; MRI: Magnetic resonance imaging; ROM: Range of motion; CT: computed tomography.

## Competing interests

The authors declare that they have no competing interests, no proprietary, no financial, no professional or other personal interest of any nature or kind in any product, service and/or company that could be construed as influencing the position presented in, or the review of, the manuscript entitled, "Clinical results of reconstruction technique using endobuttons for complete tendon Tear of pectoralis major muscle: report of five cases".

## Authors' contributions

All the authors contributed towards the clinical and surgical endeavour and in drafting of the manuscript and have given final approval of the version to be published.
